# Dehydroepiandrosterone Shifts Energy Metabolism to Increase Mitochondrial Biogenesis in Female Fertility with Advancing Age

**DOI:** 10.3390/nu13072449

**Published:** 2021-07-17

**Authors:** Chia-Jung Li, Li-Te Lin, Kuan-Hao Tsui

**Affiliations:** 1 Department of Obstetrics and Gynaecology, Kaohsiung Veterans General Hospital, Kaohsiung 813, Taiwan; nigel6761@gmail.com (C.-J.L.); litelin1982@gmail.com (L.-T.L.); 2 Institute of Biopharmaceutical Sciences, National Sun Yat-sen University, Kaohsiung 804, Taiwan; 3 Department of Obstetrics and Gynaecology, National Yang-Ming University School of Medicine, Taipei 112, Taiwan; 4 Department of Obstetrics and Gynaecology, Taipei Veterans General Hospital, Taipei 112, Taiwan; 5 Department of Pharmacy and Master Program, College of Pharmacy and Health Care, Tajen University, Pingtung County 907, Taiwan; 6 Department of Medicine, National Defense Medical Center, Tri-Service General Hospital, Taipei 114, Taiwan; 7 College of Health and Nursing, Meiho University, Pingtung County 912, Taiwan

**Keywords:** dehydroepiandrosterone, metabolic shifts, cumulus cells, infertility

## Abstract

Female reproductive aging is an irreversible process associated with a decrease in oocyte quality, which is a limiting factor for fertility. Previous studies have shown that dehydroepiandrosterone (DHEA) has been shown to improve in vitro fertilization (IVF) outcomes in older women. Herein, we showed that the decline in oocyte quality with age is accompanied by a significant decrease in the level of bioenergetic metabolism genes. We compared the clinical characteristics between groups of infertile women who either received DHEA or did not. Treatment with DHEA may enhance oocyte quality by improving energy production and metabolic reprogramming in cumulus cells (CCs) of aging women. Our results showed that compared with the group without DHEA, the group with DHEA produced a large number of day-three (D3) embryos, top-quality D3 embryos, and had improved ongoing pregnancy rate and clinical pregnancy rate. This may be because DHEA enhances the transport of oxidative phosphorylation and increases mitochondrial oxygen consumption in CCs, converting anaerobic to aerobic metabolism commonly used by aging cells to delay oocyte aging. In conclusion, our results suggest that the benefit of DHEA supplementation on IVF outcomes in aging cells is significant and that this effect may be mediated in part through the reprogramming of metabolic pathways and conversion of anaerobic to aerobic respiration.

## 1. Introduction

Since the advent of assisted reproductive technology, fertility challenges in infertile patients with ovulation disorders, endocrine abnormalities, and pelvic inflammatory diseases have been largely resolved. However, the outcome of assisted conception in older women has been unsatisfactory, and the results of studies in recent decades suggest that the main factor leading to decreased fertility in older women is oocyte aging [1]. The number of intracellular mitochondria changes with age. In women, with aging, the antioxidant capacity of the body gradually decreases, intracellular oxygen radicals decrease, reactive oxygen species (ROS) accumulate, and the mitochondria of oocytes are exposed to ROS accumulation for a long time, increasing the possibility of mitochondrial mutation and abnormal function, which further affects the quality of oocytes [2].

Oocytes require a lot of energy to maintain their cellular constancy and are, therefore, also rich in mitochondria to provide sufficient energy. However, glucose within the oocyte cannot be catabolized directly by glycolysis for energy supply, and the cytoplasmic protrusions of the cumulus cells (CCs) are in direct contact with the oocyte through the zona pellucida, while the end links the oocyte plasma membrane through a slit to provide the oocyte with nutrient metabolites [3]. Oocytes obtain amino acids, nucleotides, glutathione, and sugar metabolites [4] through slit links for energy [5]. Herrick et al. [6] found that goat cumulus–oocyte complexes (COCs) had a glycolytic rate that was three times their oxidative phosphorylation rate, that glucose was a key energy metabolite for COCs during oocyte maturation, and that glycolysis was the key energy metabolic pathway. Due to low oocyte phosphofructokinase and lactate dehydrogenase activities, the efficiency of glycolysis in oocytes is only 50% of that of COCs. Glucose is mainly produced by CC glycolysis to produce pyruvate and lactate, which are transferred to oocyte mitochondria to supply energy for its catabolism [7]. The oocyte, in turn, ensures its pyruvate supply by upregulating the expression of glycolytic genes in CCs [8]. Analysis of the expression localization of aldose reductase and sorbitol dehydrogenase in ovarian granulosa cells and oocytes showed that sorbitol, formed by the reductive dehydrogenation of glucose in granulosa cells, is indirectly synthesized by oocytes to supply fructose [9]. Thus, oocyte maturation is mainly provided with energy material by cumulus and granulosa cells.

There are many nutrients for replenishing germ cells, including supplements that activate mitochondria and enhance endocrine function. Dehydroepiandrosterone (DHEA) is the most abundant steroid in human blood circulation, an essential substrate for the synthesis of steroid hormones with many potential effects, and mainly produced in the adrenal cortex and ovarian cells of women [10]. Cholesterol is synthesized into DHEA, then catalyzed by a series of enzymes, mainly cytochrome P450, then reaches the target organ cells as DHEA sulfate (DHEA-S), which is subsequently converted into steroid hormones such as androstenedione, testosterone, and estrone by the action of steroid hormone metabolizing enzymes [11]. Previous studies have illustrated that oral DHEA 50 mg daily significantly increases free testosterone, total testosterone, androstenedione, and DHEA-S levels and that in women over 40 years of age, supplementation with 50 mg DHEA daily increases androstenedione and total testosterone levels approximately twofold [12]. Similar findings were obtained in previous studies, wherein DHEA supplementation for about 3 months in patients with poor ovarian response increased DHEA-s and total testosterone levels by 4.4-fold and threefold, respectively [13]. Epidemiological evidence suggests that serum DHEA levels in women decrease with age [14] and that this decrease is associated with increasing age as well as the onset or development of a variety of diseases, including cognitive decline, dementia, cardiovascular disease, and obesity [15,16,17].

Based on our previous studies and several studies to date, we hypothesized that DHEA supplementation may rejuvenate oocytes by activating energy metabolism within cumulus and granulosa cells, which in turn transfers energy to the oocyte. To test this hypothesis, we attempted to compare the molecular metabolic mechanisms of aging patients taking DHEA, as no association between DHEA and metabolic reprogramming has been reported. For this purpose, we recruited 77 infertile patients and used a highly sensitive energy analyzer to evaluate the unresolved issue of DHEA regulation of oocyte metabolism in energy.

## 2. Materials and Methods

### 2.1. Ethics Statement

All procedures performed in this study were approved by the Institutional Review Board of the Kaohsiung Veterans General Hospital, Kaohsiung, Taiwan (VGHKS19-CT6-17 and VGHKS14-CT10-16). This study was also registered in the Clinical Trial Register (ClinicalTrials.gov Identifier: NCT03438812). All participants signed informed consent. This study was implemented in accordance with the principles of the Declaration of Helsinki.

### 2.2. Clinical Sample and Design

**Figure 1:**
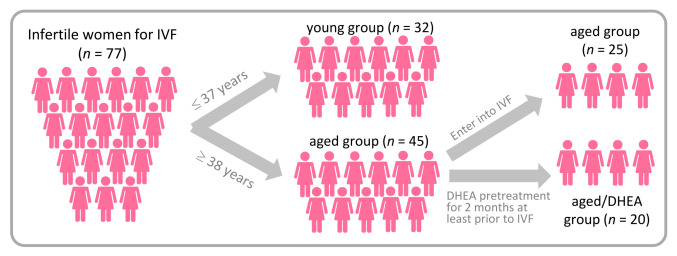
A flow chart showing the study design and selection of eligible studies.

This study was conducted at the Reproductive Center of the Kaohsiung Veterans General Hospital from June 2017 to February 2018 and subjects were recruited to participate in the study. We divided the infertile patients into a young group (age <38 years) and an aged group (age >38 years). The aged group was then randomized to receive DHEA or none. DHEA patients were given 25 mg DHEA capsules (New Dios-25 Veg capsules Toppure Biotechnology Co., Ltd., New Taipei city, Taiwan) three times daily for at least 8 weeks prior to entering their in vitro fertilization (IVF) cycle (Figure 1). The young group was set as a normal control group, the aged no-DHEA group was another control group, and the aged-DHEA group was the study group. Patients with a history of prior oophorectomy, those who received a donor cycle, those who received pelvic radiotherapy or chemotherapy, and those who received hormonal therapy within the last 3 months were excluded from this study. The clinical characteristics are shown in Table 1 and Table 2. The study was conducted in accordance with the World Medical Association Declaration of Helsinki and written informed consent was obtained from all participants.

### 2.3. Collection of CCs from Patients

The collection of CCs was performed according to previously published operational steps [18]. Briefly, after the embryologist separated the CCs from the oocytes, the CCs were mechanically disassembled, pooled, and passed through a series of centrifuges in PBS/BSA at 800× *g* for 5 min, then washed three times. The final precipitate was resuspended in Histopaque 1077 (Sigma-Aldrich, St. Louis, MO, USA) with fetal bovine serum (Gibco, Thermo Fisher Scientific, Waltham, MA, USA), insulin, transferrin, and sodium selenite (ITS, Sigma- Aldrich, St. Louis, MO, USA), and androstenedione (4-androstene-3,17-dione, Sigma). CCs were cultured at multiplate for 24 h at 37.5 °C in a humidified incubator with 5% CO_2_ for further studies.

### 2.4. RNA Extraction and Real-Time PCR

Total cellular RNA was extracted using the EasyPrep Total RNA Kit (BIOTOOLS Co., Ltd., Taipei, Taiwan) according to the manufacturer’s instructions as described previously [19]. For stable analysis of gene expression, we used the ToolScript MMLV RT kit (BIOTOOLS Co., Ltd., Taipei, Taiwan) to synthesize cDNA. q-PCR was performed using the StepOnePlusTM system (Applied Biosystems, Waltham, MA, USA) and TOOLS 2X SYBR qPCR Mix (BIOTOOLS Co., Ltd., Taipei, Taiwan). The expression levels of all genes in the cells were normalized to the internal control RNU6-1 gene. All samples with >1% variation coefficient of Ct values were retested.

### 2.5. Oxygen Consumption Rate Measurement

Oxygen consumption rate (OCR) was measured using the Seahorse XF HS mini platform extracellular flux analyzer (Agilent Technologies, Santa Clara, CA, USA). We analyzed the results according to the Seahorse bioenergetics analyzer user manual. CCs were inoculated into Seahorse XF HS 8-well plates approximately 24 h prior to the experiment according to the Seahorse protocol. After the initial experiment, the ideal density of 2000 cells per well was selected. The day before the experiment, sensor columns are hydrated in XF calibration solution and maintained at 37 °C in the air without CO_2_. On the day of the experiment, cells are washed once and incubated with a bicarbonate-free, low-buffer assay medium for 1 h at 37 °C. The cells are then incubated for 1 h at 37 °C. Changes in cellular respiration were assessed overtime during the mitochondrial function assay, and oligomycin, FCCP, and antimycin A/rotenone were sequentially administered.

### 2.6. RNA Sequencing

Total RNA was extracted from samples using TRIzol reagents according to the instructions of a previous study [20], and libraries were prepared using TruSeq Stranded mRNA LT sample preparation kits (Illumina, San Diego, CA, USA) according to the manufacturer’s instructions. Differential gene expression analysis was performed with log 2-fold change ≥1 or ≤−1, *p* < 0.05, and false discovery rate ≤5%.

### 2.7. Statistical Analysis

The data measured in this study were subjected to independent experiments at least four times, and all data in the results represent the mean ± error mean of repeated measurements. Statistical significance was assessed using GraphPad Prism 8.0 (GraphPad Software, San Diego, CA, USA), followed by Tukey post-hoc tests, using a two-way analysis of variance test to evaluate the differences between group means. Differences were considered statistically significant when *p* < 0.05.

## 3. Results

### 3.1. Basic Characteristics of Patients Undergoing IVF Cycles

A total of 77 patients, divided into young (*n* = 32), aged (*n* = 25), and aged/DHEA (*n* = 20) groups, were recruited for this study. The baseline characteristics of the three groups are shown in Table 1. There were significant differences in mean age and prior IVF failure among the three groups. In addition, there were no significant differences in BMI, duration of infertility, primary infertility, secondary infertility, basal follicle-stimulating hormone (FSH), basal E2, and basal luteinizing hormone.

### 3.2. Cycle Characteristics and Clinical Outcomes of Patients Undergoing IVF Cycles

The patient characteristics and pregnancy outcomes of the IVF cycles in the young, aged, and aged/DHEA groups are shown in Table 2. There were no significant differences in stimulation duration and gonadotropin dose between the three groups. Further analysis of clinical characteristics in the young versus aged group showed significantly higher numbers of retrieved oocytes (8.7 ± 4.1 vs. 3.2 ± 2.1), metaphase II oocytes (6.4 ± 3.2 vs. 1.8 ± 1.7), maturation rate (60.7 ± 22.1 vs. 48.1 ± 32.0), fertilized oocytes (8.3 ± 2.4 vs. 2.5 ± 1.4), number of day-three (D3) embryos (6.6 ± 3.3 vs. 1.7 ± 2.1), and number of top-quality D3 embryos (3.1 ± 2.1 vs. 0.7 ± 1.2). Similarly, this result was also reflected in the clinical pregnancy rate (53.1% vs. 16%), ongoing pregnancy rate (50% vs. 16%), and live birth rate (50% vs. 12%), which were all significantly higher in the young group than in the aged group. In the subsequent analysis of the no-DHEA aged group compared to the aged/DHEA group, there were significant increases in the number of recovered oocytes (3.2 ± 2.1 vs. 5.2 ± 1.4), number of D3 embryos (1.7 ± 2.1 vs. 3.4 ± 1.6), and number of top-quality D3 embryos (0.7 ± 1.2 vs. 2.4 ± 1.7) compared to the aged group. The no-DHEA aged group also had a significantly lower clinical pregnancy rate (16% vs. 26.3%), ongoing pregnancy rate (16% vs. 26.3%), and live birth rate (12% vs. 16.7%) than the aged/DHEA group.

### 3.3. Enrichment Analysis of Key Modules of CCs by DHEA

**Figure 2:**
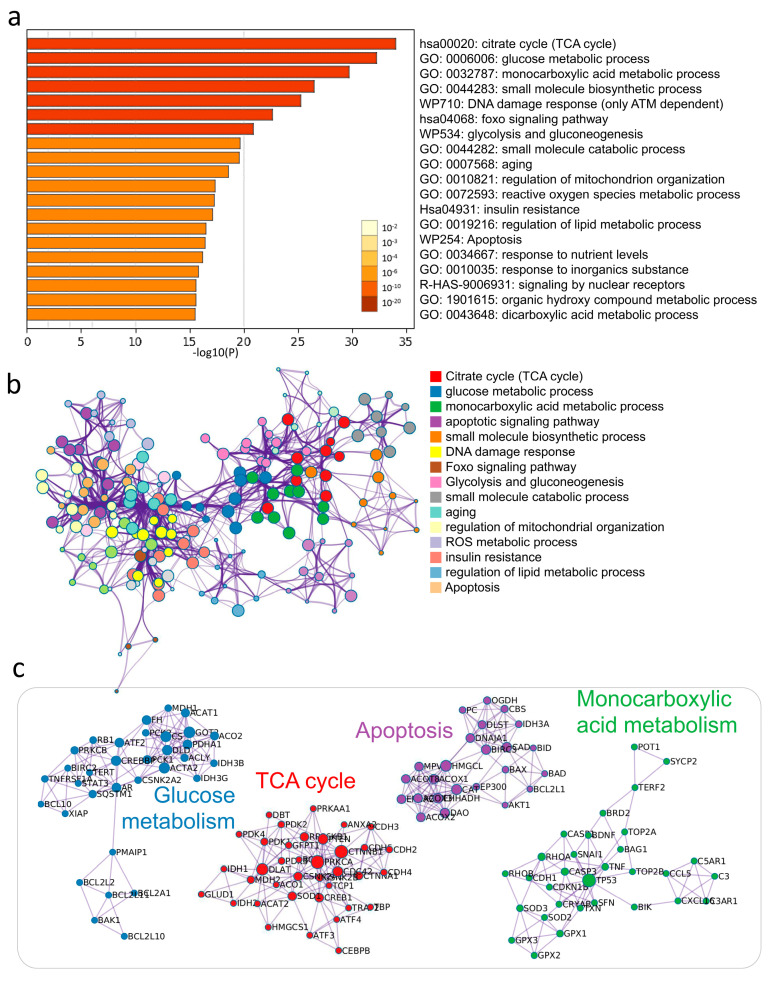
Supplementation with DHEA regulates potential metabolic pathways in the cumulus cells of elderly infertile patients. (**a**) Functional enrichment analysis of DEGs. The bar graph shows the top 20 results of the enrichment analysis of DHEA altered aging genes. (**b**) Networks of enriched protein-protein interactions for the same input list. (**c**) Biological pathways are significantly enriched in aging CCs response genes regulated by DHEA.

To investigate the biological significance of DHEA affecting CCs, we screened 306 genes with more significant alterations after passing the samples through NGS and uploaded them to Metascape software (Metascape Ltd., London, England) for functional enrichment analysis. The Metascape analysis revealed the top 20 enrichment clusters (Figure 2a, Supplementary Table S1). To understand the possible molecular pathways and interactions of DHEA potentially regulating CCs, we performed a meta-landscape analysis using the Metascape tool suite to filter the higher scoring markers among the differential genes in aged and aged/DHEA and enriched the molecular pathways of these genes with protein–protein interaction (PPI) enrichment (Figure 2b). We then selected a subset of representative terms from this cluster and transformed them into network layouts. More specifically, each term was represented by a round node whose size is proportional to the number of input genes falling into the term, and whose color represents its cluster identity. To confirm that DHEA regulates potential PPI complexes, we compiled the molecular interactors in each screen into a single list of genes and generated a merged PPI network (Figure 2c). The network contained a subset of proteins that form physical interactions with at least one other member of the list based on the established database of interactions. We applied a molecular complex detection algorithm to identify the densely connected network components. We observed that PPI clusters involved many signaling pathways and, most significantly, enriched the interactions regarding several different glucose metabolism pathways, TCA cycle, apoptosis, and monocarboxylic acid metabolism.

### 3.4. DHEA Regulates Energy Production Pathways in Aging Cells

**Figure 3:**
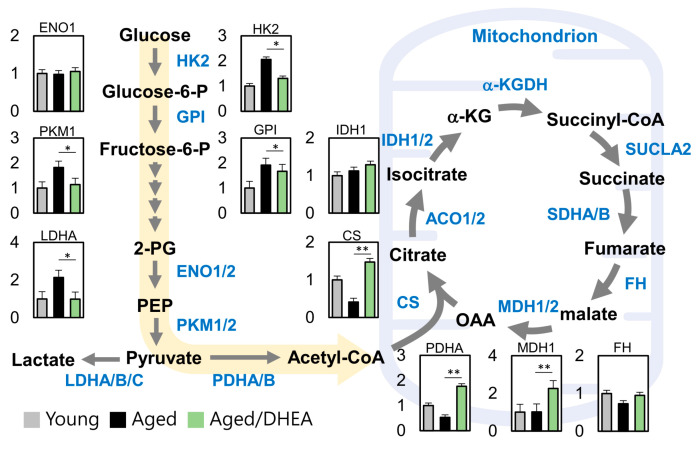
Metabolic reprogramming in the cumulus cells of aged infertile patients after DHEA treatment. A map of metabolic pathways and metabolite levels in glycolysis and TCA cycle pathways. * *p* <0.05 and ** *p* < 0.01.

To determine the DHEA-regulated metabolic pathway in aged cells, we analyzed the energy metabolism genes in the three groups, namely young, aged, and aged/DHEA. We found significant differences between cells in the number of metabolites originating from glycolysis and the TCA cycle. Further, pyruvate regulated the key gene of acetyl-CoA, PDHA, which was significantly elevated in the DHEA group compared to the aged group, and further regulated CS and MDH1. Further, pyruvate regulated high levels of CS and MDH1. Collectively, these data suggest that DHEA induces changes in glucose metabolism and the TCA cycle in aging CCs (Figure 3).

### 3.5. DHEA Normalizes the Decrease in the Oxygen Consumption Rate of Aged CCs

**Figure 4:**
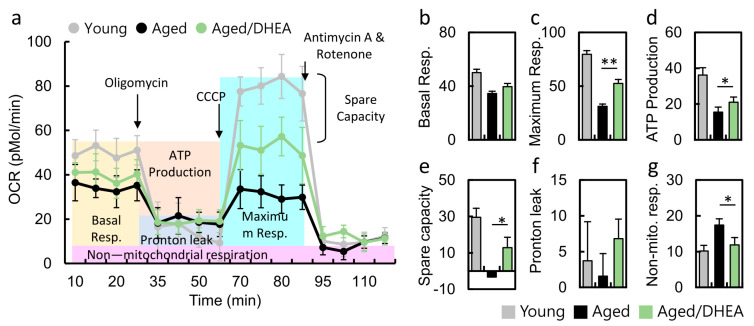
Metabolic reprogramming in the cumulus cells of aged infertile patients after DHEA treatment. (a) Mitochondrial function was determined by oxygen consumption rate (OCR), through utilizing the Seahorse Bioscience Analyzer. (b-g) All of the OCR values at different phases of basal respiration, maximal respiration, ATP-coupled respiration, and spare capacity, proton leak and noon-mitochondrial respiration were recorded, and the values were calculated among the young, aged and aged/DHEA groups. Oligomycin (1 uM), FCCP (1 uM), antimycin (0.5 uM), rotenone (0.5 uM). * *p* < 0.05 and ** *p* < 0.01.

The real-time measurement of oxygen consumption rate is a direct measurement of cell mitochondrial function under physiological conditions. Treatment with oligomycin (an ATP synthase inhibitor) revealed the amount of oxygen consumption required for ATP synthesis. FCCP is a non-coupling agent that induces membrane potential dissipation and rapid oxygen consumption and is therefore defined as the maximum respiratory capacity of mitochondria. Antimycin A and rotenone are mitochondrial complex I and III inhibitors that completely block mitochondrial respiration, demonstrating non-mitochondrial respiration (Figure 4a). Thus, the OCR curves in Figure 4a show a significant decrease in aging CCs compared to younger CCs. Cells from patients taking DHEA regained normal cellular respiration, including maximum OCR (Figure 4c), ATP conversion rate (Figure 4d), and spare capacity (Figure 4e). Moreover, non-mitochondrial respiration was significantly lower in the DHEA group than in the no-DHEA aged group. Respiratory reserve capacity represents the reserve capacity of cells to produce ATP through oxidative phosphorylation when energy demand increases. These findings suggest that DHEA administration may modulate cellular mitochondria, increasing the mitochondrial turnover rate to increase intracellular energy.

## 4. Discussion

The important regulatory mechanisms of DHEA to improve IVF treatment in infertile patients are mainly the following: 

Increasing the effect of FSH on the ovary: Previous studies have shown that in the ovary, DHEA increases the sensitivity of granulosa cells to gonadotropins such as FSH, promotes follicle recruitment, and increases the number of follicles that can be recruited in the ovary [21].

Regulation of the androgen receptor (AR) pathway: In the ovary, androgens act synergistically with the AR to regulate the initiation, growth and development, atresia and apoptosis, and ovulation of the female follicle. Supplementation with DHEA may promote the biological function of androgen-related signaling pathways by upregulating AR expression or by directly interacting with the AR. DHEA supplementation may promote the biological function of the androgen-related signal transduction pathway and participate in follicle recruitment and growth [22].

Increasing IGF-1 levels: IGF-1 is an important factor in the normal reproductive function of the ovary and is involved in the regulation of follicle development through local autocrine and paracrine forms. Supplementation with DHEA increases IGF-I levels in the ovary, which in turn has a positive effect on follicle growth and oocyte quality [23].

Increasing AMH levels: DHEA supplementation increases the androgen content within follicles, which in turn induces an increase in AMH and inhibin B. This reduces the sensitivity of follicles to FSH and affects follicular growth and development; it inhibits primordial follicle recruitment and prevents premature and rapid depletion of primordial follicles. Nielsen et al. showed that measuring serum AMH levels predicted pregnancy outcomes in women receiving DHEA pretreatment, and when serum AMH levels were >1.05 ng/mL, the clinical pregnancy and live birth rates were significantly increased, and the miscarriage rate was significantly improved [24].

Improvement of the ovarian microenvironment: While a gradual decline in the number of remaining follicles with age is inevitable, the decline in oocyte quality with age is controversial. Previous studies have shown that age-related embryonic aneuploidy is significantly reduced with DHEA supplementation [25].

Mitochondrial dysfunction is associated with reproductive decline in humans and has implications for IVF. Mitochondrial nutrients are a class of biological or chemical complexes that promote energy production within mitochondria. α-lipoic acid is an important coenzyme for mitochondrial metabolism and is also essential for mouse embryonic survival [26]. Resveratrol is an anti-aging compound that can improve the mitochondrial number and mitochondrial function [27]. The addition of L-carnitine supplementation in the cytoplasm of mouse oocytes may contribute to the formation of a spindle during oocyte maturation in vitro, spindle formation, and normal mitochondrial distribution during in vitro maturation [28]. Coenzyme Q10 is a lipid-soluble electron transfer agent whose levels decrease with age, and studies have reported a shift to higher levels of Q10. Coenzyme Q10 supplementation in aged mice has been shown in the literature to delay the reduction in ovarian reserve, repair ovarian mitochondrial gene expression, and improve mitochondrial activity [29]. Although the use of mitochondrial nutrients sounds attractive, its clinical effects need to be further investigated.

Our team has been investigating the mitochondrial molecular regulation of DHEA in granulosa and cumulus cells. In this study, we found that DHEA has a reprogrammed metabolic pathway and further activates mitochondrial oxidative phosphorylation. This result is also consistent with previous studies stating that DHEA protects human granulosa cells from the dual-mode of apoptosis and necroptosis involving mitochondria [30]. We had previously analyzed the application of DHEA for enhancing mitochondrial function and reducing CC apoptosis in infertile patients. DHEA also ameliorated the abnormal mitochondrial dynamics and mitophagy of CCs in poor ovarian responders [31,32]. In addition, we found that DHEA can slow down the progression of aged CCs [33]. However, it has been suggested that DHEA activates the activity of CREB1, a key transcription factor for energy metabolism, which in turn regulates downstream biogenesis-related genes such as PGC1*α* and TFAM [34]. Therefore, this study seems to suggest that DHEA potentially regulates biogenesis key transcription factors, which in turn affects the intracellular energy metabolic pathway, and that energy-rich cells can also reduce the programmed cell death caused by aging, such as mitophagy, necroptosis, and apoptosis. An important limitation of our study was its small sample size. Therefore, a trend of increase was observed in clinical outcomes, but it was not significant. Thus, we should interpret the data carefully.

## 5. Conclusions

**Figure 5:**
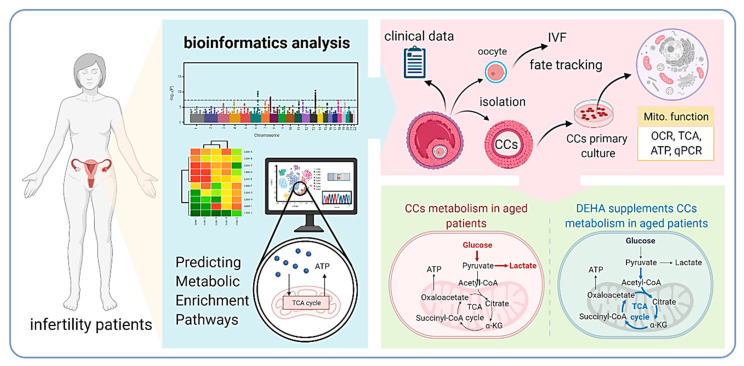
Proposed model of DHEA regulation of metabolic pathways in infertile patients.

This study suggested that DHEA supplementation altered the levels of glycolysis genes and increased mitochondrial oxidative phosphorylation, thereby enhancing the energy metabolism of aging cells and elevating pregnancy rates in infertile patients. Our observations may provide a possible rationale for the clinical use of DHEA supplementation in patients undergoing IVF (Figure 5).

## Figures and Tables

**Table 1:** Basic characteristics of patients in the young, aged, and aged/DHEA groups.

Parameters	Young (≤37) (*n* = 32)	Aged (≥38) (*n* = 25)	Aged/DHEA (*n* = 20)
Age (years)	35.7 ± 4.1	41.3 ± 3.4 *	40.8 ± 2.7 *
BMI (kg/m^2^)	22.7 ± 2.6	23.1 ± 2.9	24.7 ± 3.1
Duration of infertility (years)	3.6 ± 1.9	2.8 ± 2.4	3.0 ± 3.5
Previous IVF failure (*n*)	1.2 ± 1.4	1.4 ± 2.1	3.4 ± 2.7 *^#^
Types of infertility *n* (%)			
Primary infertility	16/32 (50%)	12/25 (48%)	10/20 (50%)
Secondary infertility	18/32 (56%)	12/25 (48%)	9/20 (45%)
Basal FSH (IU/l)	4.7 ± 2.5	5.8 ± 5.1	5.5 ± 4.2
Basal E2 (pg/mL)	91.2 ± 67.5	107.1 ± 62.8	94.6 ± 77.2
Basal LH (IU/l)	4.9 ± 2.8	4.2 ± 3.3	5.3 ± 4.3

DHEA, dehydroepiandrosterone; IVF, in vitro fertilization; FSH, follicle stimulation hormone; E2; Estradiol; LH, *luteinizing* hormone. * *p* < 0.05 versus young; # *p* < 0.05 versus aged.

**Table 2:** Cycle characteristics and pregnancy outcome in the young, aged, and aged/DHEA groups.

Parameters	Young (*n* = 32)	Aged (*n* = 25)	Aged/DHEA (*n* = 20)
Stimulation duration (days)	10.2 ± 1.1	10.7 ± 2.9	10.4 ± 1.8
HMG/FSH dose (IU)	3040.3 ± 660.1	2836.3 ± 812.2	2982.1 ± 604.1
No. of oocytes retrieved (*n*)	8.7 ± 4.1	3.2 ± 2.1 *	5.2 ± 1.4 *^#^
No. of metaphase II oocytes (*n*)	6.4 ± 3.2	1.8 ± 1.7 *	2.3 ± 1.5 *
Maturation rate (%)	60.7 ± 22.1	48.1 ± 32.0 *	62.5 ± 42.1
No. of fertilized oocytes (*n*)	8.3 ± 2.4	2.5 ± 1.4 *	2.8 ± 1.9 *
Fertilization rate (%)	71.3 ± 18.6	67.8 ± 21.2	71.7 ± 22.0
No. of Day 3 embryos (*n*)	6.6 ± 3.3	1.7 ± 2.1 *	3.4 ± 1.6 *^#^
No. of top-quality D3 embryos (*n*)	3.1 ± 2.1	0.7 ± 1.2 *	2.4 ± 1.7 *^#^
Clinical pregnancy rate % (*n*)	53.1% (17/32)	16.0% (4/25)	26.3% (6/20)
Ongoing pregnancy rate % (*n*)	50.0% (16/32)	16.0% (4/25)	26.3% (6/20)
Live birth rate % (*n*)	50.0% (16/32)	12.0% (3/25)	16.7% (4/20)

DHEA, dehydroepiandrosterone; HMG, human menopausal gonadotrophin; FSH, follicle stimulation hormone. * *p* < 0.05 versus young; # *p* < 0.05 versus aged.

## Data Availability

Not applicable.

## Supplementary Materials

The following are available online at https://www.mdpi.com/article/10.3390/nu13072449/s1, Table S1: Enrichment analysis of key modules of CCs in infertility patients affected by DHEA.
