# Supplementary material for: Dehydroepiandrosterone Shifts Energy Metabolism to Increase Mitochondrial Biogenesis in Female Fertility with Advancing Age
Source: Nutrients. 2021 Jul 17;13(7):2449. doi: 10.3390/nu13072449 (PMC8308577; doi:10.3390/nu13072449)
Supplement: Supplementary file 1 [file nutrients-13-02449-s001.zip › nutrients-1270594-supplementary.pdf]

Supplementary table S1: Enrichment analysis of key modules of CCs in infertility patients affected by DHEA.

GO	Category	Description	Count	%	Log10(P)	Log10(q)
hsa00020	KEGG Pathway	Citrate cycle (TCA cycle)	20	8.10	-34.04	-29.98
GO:0006006	GO Biological Processes	glucose metabolic process	34	13.77	-32.27	-28.61
GO:0032787	GO Biological Processes	monocarboxylic acid metabolic process	48	19.43	-29.68	-26.10
GO:0097190	GO Biological Processes	apoptotic signaling pathway	43	17.41	-26.44	-23.16
GO:0044283	GO Biological Processes	small molecule biosynthetic process	45	18.22	-25.29	-22.10
WP710	WikiPathways	DNA Damage Response	22	8.91	-22.63	-19.57
hsa04068	KEGG Pathway	foxo signaling pathway	22	8.91	-20.87	-17.85
WP534	WikiPathways	Glycolysis and Gluconeogenesis	15	6.07	-19.60	-16.63
GO:0044282	GO Biological Processes	small molecule catabolic process	32	12.96	-19.56	-16.60
GO:0007568	GO Biological Processes	aging	27	10.93	-18.60	-15.76
GO:0010821	GO Biological Processes	regulation of mitochondrion organization	20	8.10	-17.30	-14.61
GO:0072593	GO Biological Processes	ROS metabolic process	25	10.12	-17.27	-14.59
hsa04931	KEGG Pathway	insulin resistance	18	7.29	-17.09	-14.43
GO:0019216	GO Biological Processes	regulation of lipid metabolic process	28	11.34	-16.49	-13.97
WP254	WikiPathways	Apoptosis	16	6.48	-16.38	-13.86
GO:0031667	GO Biological Processes	response to nutrient levels	29	11.74	-16.15	-13.64
GO:0010035	GO Biological Processes	response to inorganic substance	31	12.55	-15.82	-13.35
R-HSA-9006931	Reactome Gene Sets	Signaling by Nuclear Receptors	24	9.72	-15.57	-13.12
GO:1901615	GO Biological Processes	organic hydroxy compound metabolic process	31	12.55	-15.54	-13.10
GO:0043648	GO Biological Processes	dicarboxylic acid metabolic process	16	6.48	-15.50	-13.09
